# Supplementary figures and images for: MicroRNAs Influence the Migratory Ability of Human Umbilical Vein Endothelial Cells
Source: Genes (Basel). 2022 Apr 2;13(4):640. doi: 10.3390/genes13040640 (PMC9029696; doi:10.3390/genes13040640)

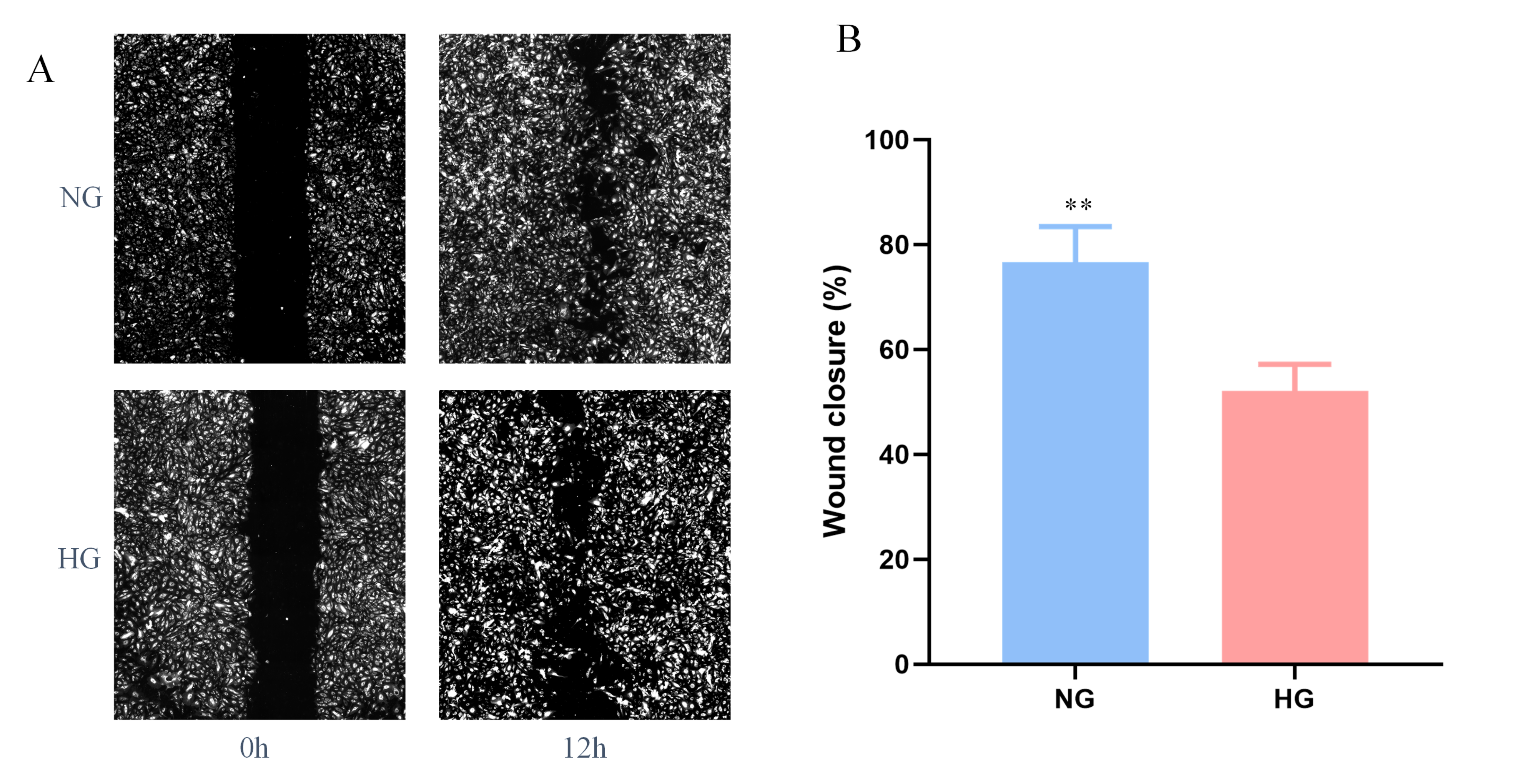

Supplement: Supplementary file 1 [file genes-13-00640-s001.zip › Supplementary Figure S1.tif]

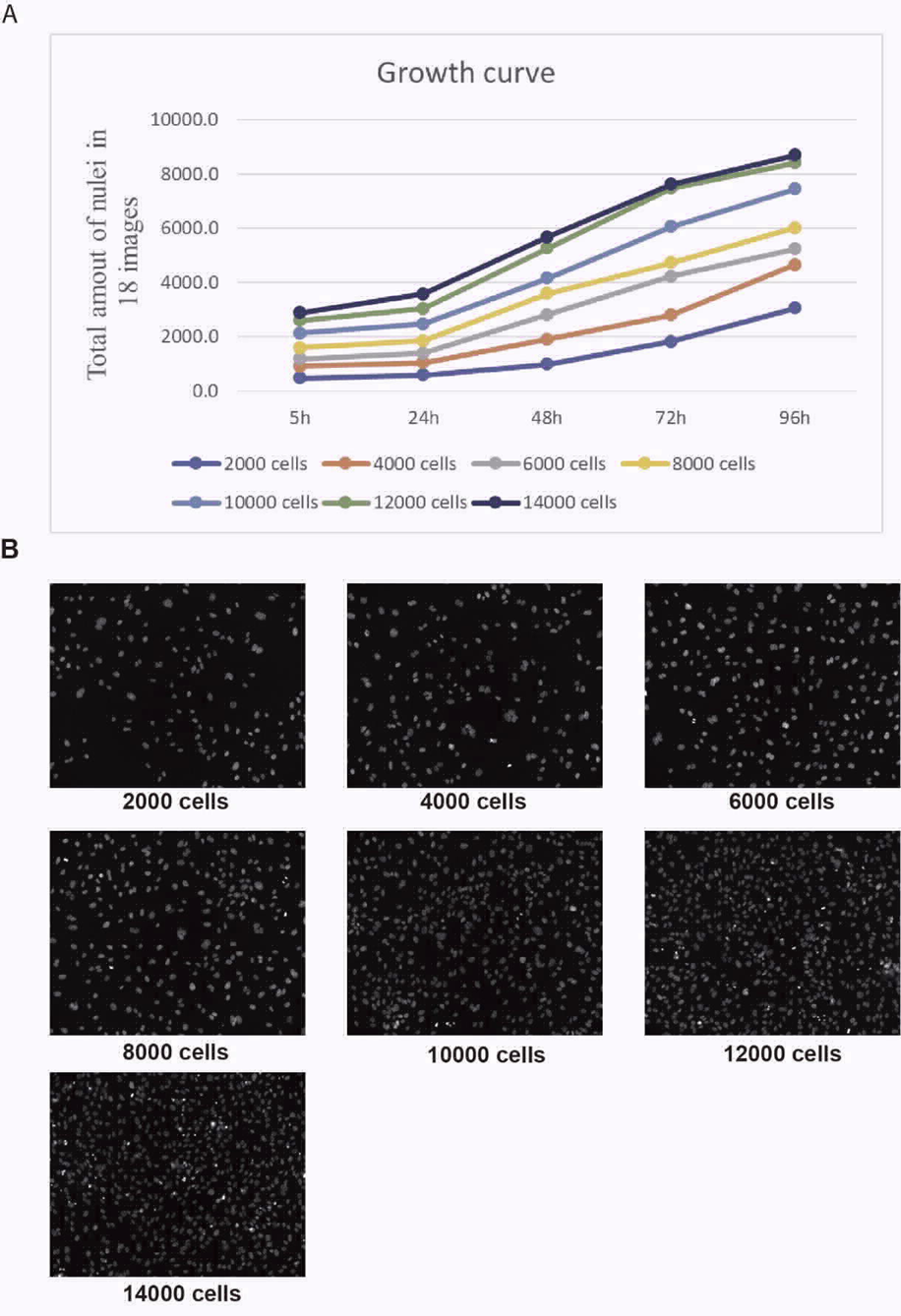

Supplement: Supplementary file 1 [file genes-13-00640-s001.zip › Supplementary Figure S2.tif]

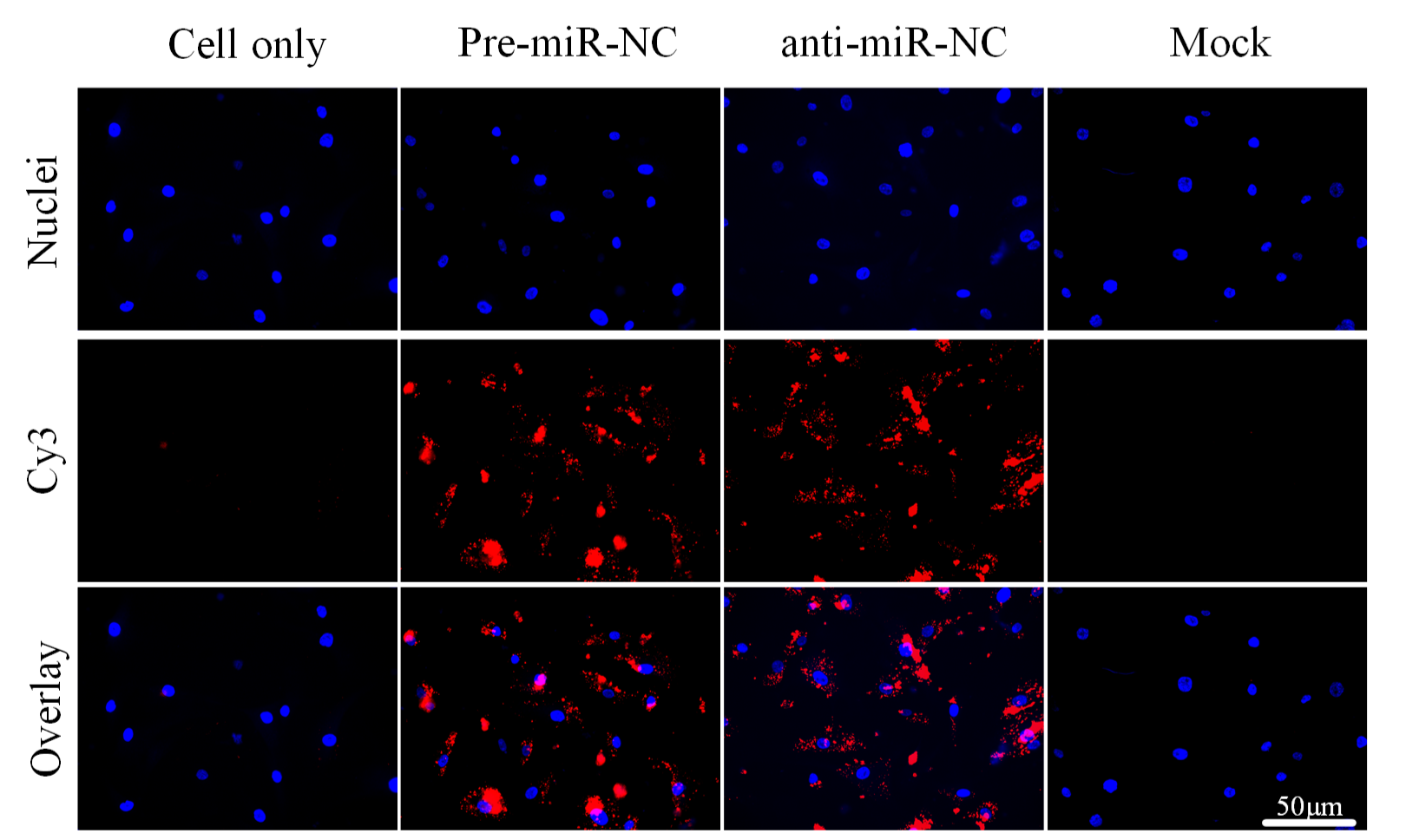

Supplement: Supplementary file 1 [file genes-13-00640-s001.zip › Supplementary Figure S3.tif]

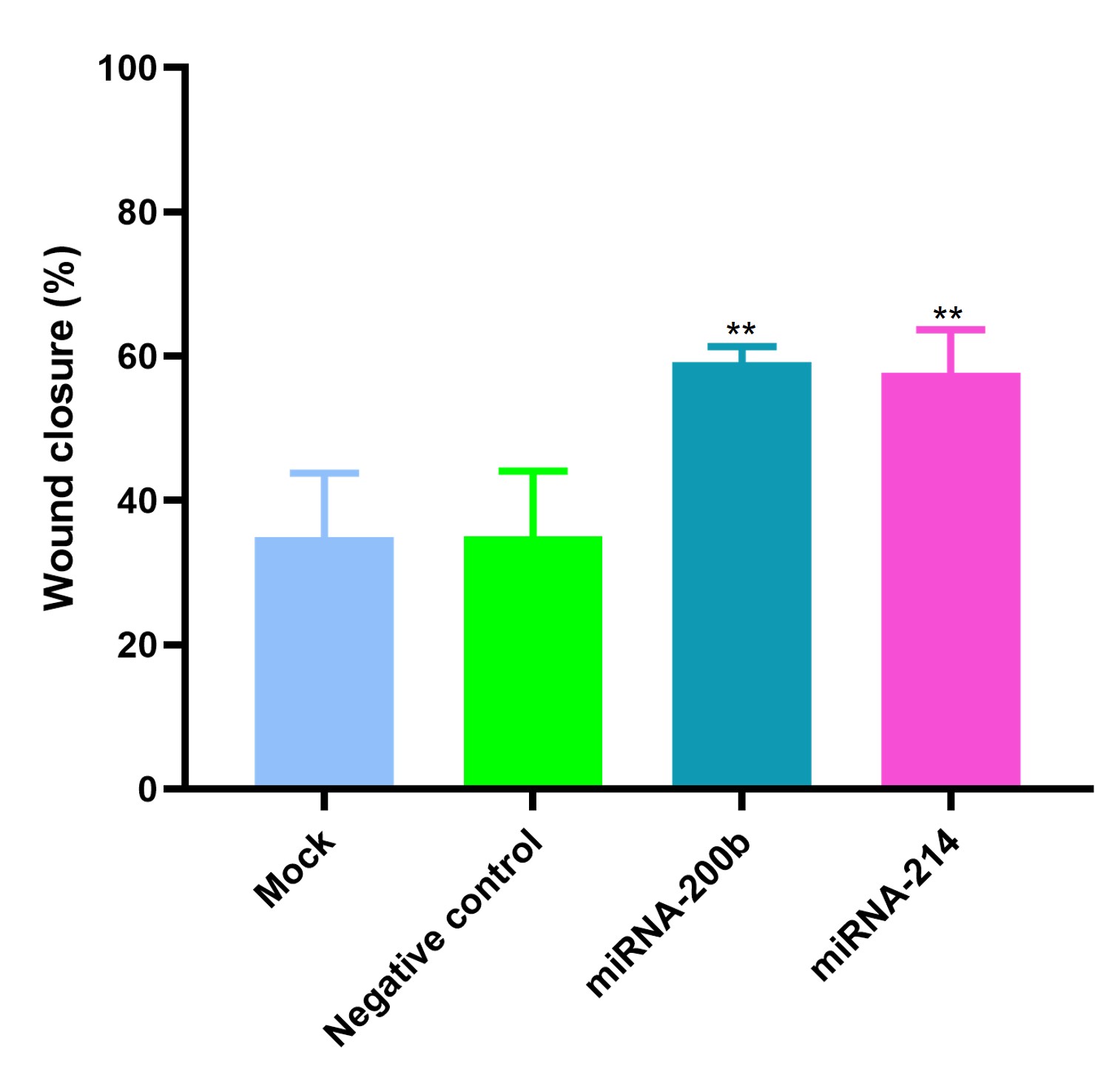

Supplement: Supplementary file 1 [file genes-13-00640-s001.zip › Supplementary Figure S4.jpg]
